# BAAV Mediated GJB2 Gene Transfer Restores Gap Junction Coupling in Cochlear Organotypic Cultures from Deaf Cx26Sox10Cre Mice

**DOI:** 10.1371/journal.pone.0023279

**Published:** 2011-08-18

**Authors:** Giulia Crispino, Giovanni Di Pasquale, Pietro Scimemi, Laura Rodriguez, Fabian Galindo Ramirez, Romolo Daniele De Siati, Rosa Maria Santarelli, Edoardo Arslan, Mario Bortolozzi, John A. Chiorini, Fabio Mammano

**Affiliations:** 1 Fondazione per la Ricerca Biomedica Avanzata, Istituto Veneto di Medicina Molecolare, Padova, Italy; 2 Dipartimento di Fisica “G. Galilei”, Università di Padova, Padova, Italy; 3 Molecular Physiology and Therapeutics Branch, National Institute of Dental and Craniofacial Research, National Institutes of Health, Bethesda, Maryland, United States of America; 4 Dipartimento di Specialità Medico–Chirurgiche e Servizio di Audiologia, Università di Padova, Padova, Italy; 5 Istituto CNR di Neuroscienze, Padova, Italy; Texas A&M University, United States of America

## Abstract

The deafness locus DFNB1 contains GJB2, the gene encoding connexin26 and GJB6, encoding connexin30, which appear to be coordinately regulated in the inner ear. In this work, we investigated the expression and function of connexin26 and connexin30 from postnatal day 5 to adult age in double transgenic Cx26*^Sox10Cre^* mice, which we obtained by crossing connexin26 floxed mice with a deleter Sox10–Cre line. Cx26*^Sox10Cre^* mice presented with complete connexin26 ablation in the epithelial gap junction network of the cochlea, whereas connexin30 expression was developmentally delayed; immunolabeling patterns for both connexins were normal in the cochlear lateral wall. In vivo electrophysiological measurements in Cx26^Sox10Cre^ mice revealed profound hearing loss accompanied by reduction of endocochlear potential, and functional experiments performed in postnatal cochlear organotypic cultures showed impaired gap junction coupling. Transduction of these cultures with a bovine adeno associated virus vector restored connexin26 protein expression and rescued gap junction coupling. These results suggest that restoration of normal connexin levels by gene delivery via recombinant adeno associated virus could be a way to rescue hearing function in DFNB1 mouse models and, in future, lead to the development of therapeutic interventions in humans.

## Introduction

Connexins are tetraspan transmembrane proteins that form hexameric assemblies in the plasma membrane called connexons; head–to–head docking of two connexons in adjacent cells establishes intercellular channels that cluster into a plaque, and the two adjoining plasma membranes in the plaque remain separated by a narrow extracellular gap of 2–3 nm [1].

GJB2, the gene encoding connexin26 (Cx26) was the first gene to be linked to an autosomal *recessive* form of deafness, DFNB1 [2], as well as to a rare *dominant* form of deafness, DFNA3 [3]. More than 90 distinct recessive mutations of GJB2 have been described, including nonsense, missense, splicing, frame–shift mutations and inframe deletions [4] (see also http://davinci.crg.es/deafness/index.php). Altogether these mutations account for approximately 50% of congenital, recessively inherited, sensorineural nonsyndromic hearing loss in several populations, with approximate carrier frequency of 1 in 33 and up to 1 in 28 amongst Mediterraneans [5] (see also http://hereditaryhearingloss.org/). DFNB1–linked familial cases with no mutation in GJB2 have also been reported and shown to be associated with two large deletions occurring upstream of GJB2 in GJB6, the gene encoding connexin30 (Cx30) which lies 30 kb telomeric to GJB2 on human chromosome 13 (chromosome 14 in the mouse) [4]. To date, a threonine–to–methionine substitution at position 5 is the only Cx30 mutation (Cx30T5M) associated to DFNA3 [6].

The recent 3.5–Å crystal structure of the wild–type human Cx26 provides the most detailed model so far available for a connexin channel [7]. Cx26 shares 77% amino acid similarity with Cx30 and both are highly expressed in non–sensory cells of the inner ear [8], [9] where they form two separate intercellular gap junction networks [10]. In the murine cochlea, the *epithelial* gap junction network forms around embryonic day 16 and connects all supporting cells in the sensory epithelium (which comprises the organ of Corti) as well as adjacent epithelial cells, and also includes interdental cells of the spiral limbus and root cells in the spiral ligament. The *connective tissue* gap junction network starts to develop around birth and comprises fibrocytes of the spiral limbus, fibrocytes of the spiral ligament as well as basal and intermediate cells of the stria vascularis, a structure responsible for K^+^ secretion and generation of the endocochlear potential [11] (the electrical potential difference between the endolymphatic and perilymphatic compartments of the cochlea which in mice appears around postnatal (P) day 5 (P5) and increases progressively to reach adult levels in excess of +100 mV by P18 [12]).

Mouse models confirmed that Cx26 and Cx30 are essential for auditory function [13] and have helped establishing a link between inherited deafness, connexin expression, endolymphatic K^+^ concentration, endocochlear potential [14], [15], [16], transfer of nutrients within the sensory epithelium of the inner ear [17] and cellular degeneration in the cochlea [18].

In this study we examine a mouse model with targeted deletion of Cx26 in the inner ear, referred to as Cx26*^Sox10Cre^* [19], obtained by crossing Cx26*^loxP/loxP^* mice, carrying the floxed GJB2 gene [14], and Sox10Cre mice, which express a Cre recombinase under the Sox10 promoter [20]. Cx26*^Sox10Cre^* mice may be a model for many DFNB1–affected patients since the most frequent GJB2 mutation, 35delG, is a single base deletion that results in a frameshift at the 12th amino acid and premature termination of the Cx26 protein [4]. We then exploited the efficient gene transfer activity and minimal toxicity of bovine adeno-associated viral (BAAV) vectors to deliver a replacement Cx26 gene and restore gap junction coupling in cochlear non–sensory cells maintained in organotypic cultures.

## Results

### Hearing impairment, reduced endocochlear potential and hair cell loss in Cx26*^Sox10Cre^* mice

Loss of Cx26 in mammary epithelium during early pregnancy results in unscheduled apoptosis and impaired development [21]. For this reason, Cx26 full knock out mice present with a lethal phenotype, calling for conditional knock out mice in which Cx26 deletion can be both controlled and specific for different tissues. By crossing Cx26*^loxP/loxP^* mice [14] with Sox10Cre mice [20], [22], we generated double transgenic Cx26*^Sox10Cre^* mice with a predicted ablation pattern of GJB2 in cells deriving from the neural crest and otic vesicle [23].

**Figure 1:**
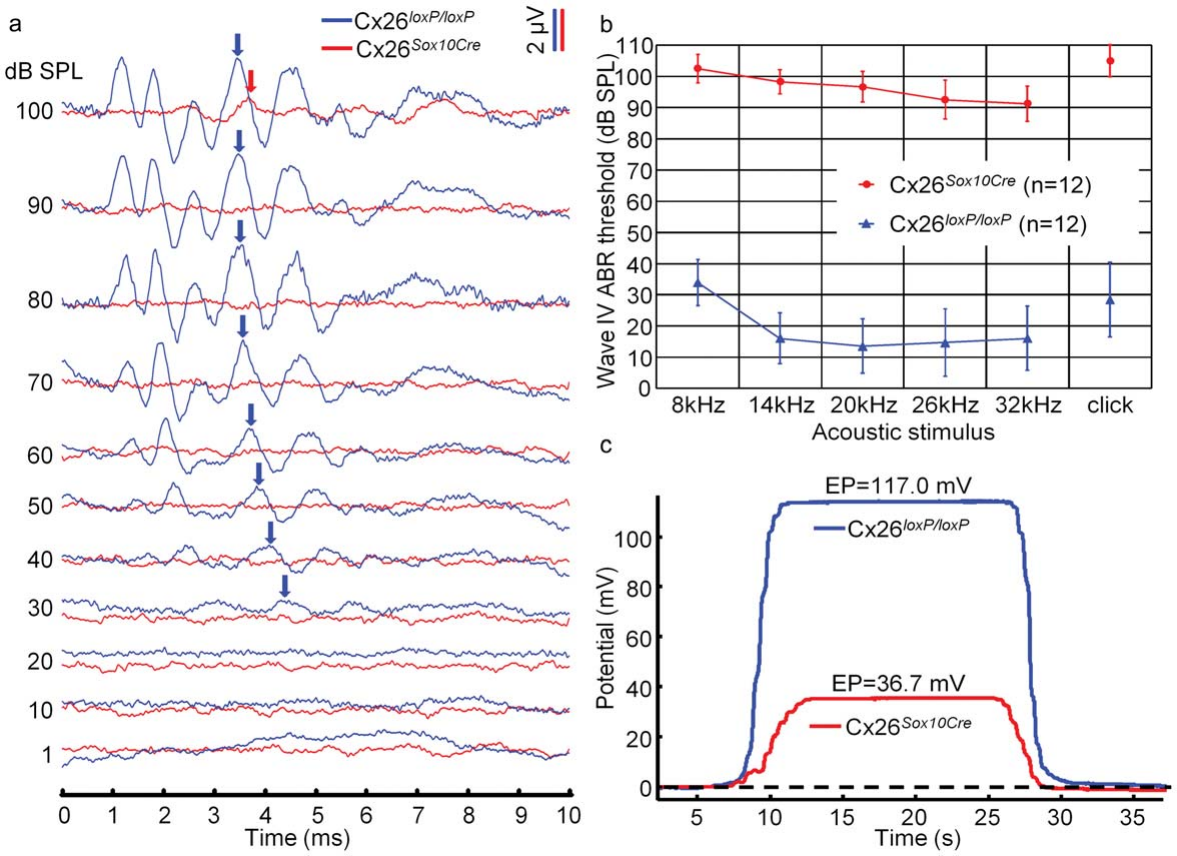
In vivo electrophysiological recordings from Cx26*^loxP/loxP^* and Cx26*^Sox10Cre^* mice. (**a**) Representative recordings of auditory brainstem responses (ABR) evoked by 14 kHz tone burst stimuli in a Cx26*^loxP/loxP^* mouse (P32, blue line) and a Cx26*^Sox10Cre^* mouse (P39, red line). Note that waves II, IV and V were detected down to 30 dB SPL in the Cx26*^loxP/loxP^* mouse, whereas no evoked responses were identified for intensities ≤90 dB SPL in the Cx26*^Sox10Cre^* mouse. (**b**) ABR audiograms for tone bursts at 8, 14, 20, 26, 32 kHz and for click stimuli obtained from Cx26*^loxP/loxP^* mice (blue line, n = 12) and Cx26*^Sox10Cre^* mice (red line, n = 12) aged between P29 and P64; error bars represent standard deviation. Note that click responses are plotted at an arbitrary point on the frequency axis (the position does not reflect the frequency content of click stimuli). (**c**) Representative recordings of endocochlear potential (EP) obtained from a Cx26*^loxP/loxP^* mouse (blue line) and a Cx26*^Sox10Cre^* (red line) mouse, aged P38 and P41 respectively. Standards for reliable recording were: 1) stable EP during minimum 10 seconds and 2) a maximum difference of ±2 mV between starting and final baseline potentials.

**Figure 2:**
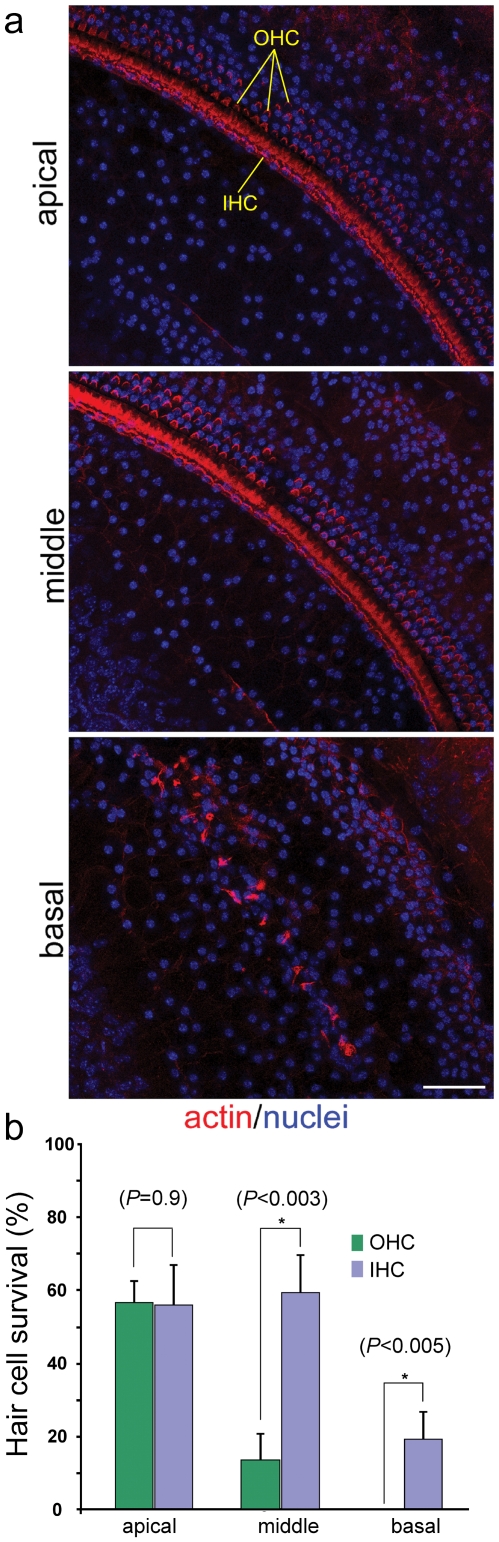
Hair cell loss in Cx26*^Sox10Cre^* mice at P30. (**a**) Horizontal sections (orthogonal to the modiolus) of cochleae from P30 Cx26^Sox10Cre^ mice. Images from apical, medial and basal turns were obtained by maximal intensity back–projection of 20 confocal optical sections from a 2 µm step though–focus sequence (z–stack). Actin filaments were stained with Texas red conjugated phalloidin (red) and nuclei with DAPI (blue); OHC, outer hair cells; IHC, inner hair cell. scale bar: 50 µm. (**b**) Percentage of hair cell survival in the basal, middle and apical turn of cochleae from Cx26*^Sox10Cre^* mice relative to corresponding controls in Cx26*^loxP/loxP^* mice.

Auditory function in Cx26*^Sox10Cre^* mice and in Cx26*^loxP/loxP^* mice, taken as controls, was quantified by recording auditory brainstem responses (ABR) which are electrical signals evoked from the brainstem following the presentation of sound stimuli (**Figure 1a**). We measured the IV wave thresholds of the ABR for click and tone burst stimuli of 8, 14, 20, 26, 32 kHz in Cx26*^loxP/loxP^* mice (n = 12) and Cx26*^Sox10Cre^* mice (n = 12) aged between P29 and P64 (**Figure 1b**). Compared to Cx26*^loxP/loxP^* mice, thresholds were significantly elevated in Cx26*^Sox10Cre^* mice (*P*<0.001, ANOVA) and in excess of 90 dB sound pressure level (SPL) for tone bursts as well as for click stimuli, whereas endocochlear potential (**Figure 1c**) was significantly reduced (38±2 mV in Cx26*^Sox10Cre^* mice, *n* = 7, vs. 109±3 mV in Cx26*^loxP/loxP^* mice, n = 11; *P*<0.001, ANOVA). These differences were paralleled by degeneration of the sensory epithelium in the basal turn of P30 Cx26*^Sox10Cre^* cochleae, affecting both sensory and non–sensory cells (**Figure 2**). The percentage of surviving hair cells was very low in the basal turn, and increased towards the apical turn (**Figure 2a**); cell loss was less dramatic for inner hair cells than for outer hair cells, which were missing altogether in the basal turn (**Figure 2b**).

### Time course of connexin immunolabeling and organ of Corti morphology

**Figure 3:**
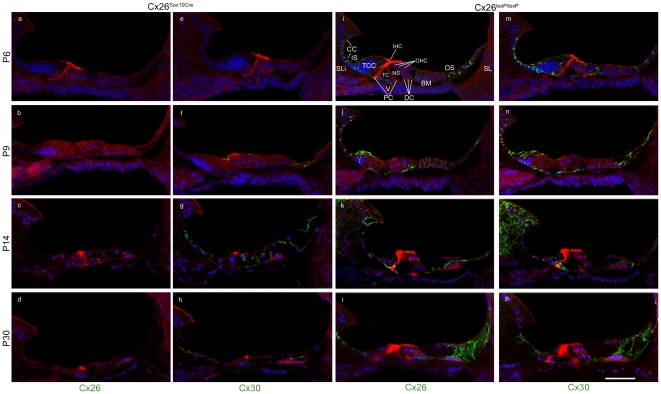
Time course of connexin immunoreactivity in the sensory epithelium of Cx26*^Sox10Cre^* mice. Maximal projection rendering of two consecutive midmodiolar confocal optical sections taken at 1 µm intervals in the basal cochlear turn of Cx26*^Sox10Cre^* mice (panels a–h) and control Cx26*^loxP/loxP^* mice (panels i–p) at P6, P9, P14 and P30. Expression of Cx26 (panels a–d, i–l) and Cx30 (panels e–h, m–p) was detected with selective antibodies (green) nuclei were stained with DAPI (blue) and actin filaments with Texas red conjugated phalloidin (red). TCC, tall columnar cells forming a transient structure, also known as Kölliker's organ; CC, cuboidal cells that replace TCC during the first two postnatal weeks; DC, Deiters' cells (also known as outer phalangeal cells); IHC, inner hair cell; IS, inner sulcus; SLi, spiral limbus; OHC, outer hair cells; OS, outer sulcus; PC, pillar cells forming the tunnel of Corti (TC); NS, Nuel's space; V, vas spiralis; BM, basilar membrane; SL, spiral ligament; scale bar, 50 µm.

To characterize connexin expression, we performed immunolabeling in the basal turn of the cochlea with antibodies specific for Cx26 or Cx30 proteins at different time points: P6, P9, P14 and P30. Inner hair cells and outer hair cells showed no sign of immunoreactivity to these antibodies, in accord with the notion that sensory cells are not coupled by gap junction channels to any other cell type in the organ of Corti [10]. Cx26 was not detected in the sensory epithelium of Cx26*^Sox10Cre^* mice at any time point (**Figure 3a–d**) whereas Cx30 was downregulated at P6 (**Figure 3e**) but its expression level started to increase between P6 and P9 (**Figure 3f**). It appeared virtually normal around P14 (**Figure 3g**) but severely deficient by P30 (**Figure 3h**) reflecting cell death in the sensory epithelium of Cx26*^Sox10Cre^* mice (**Figure 3d,h** and **Figure 2**). Morphologically, the tunnel of Corti and Nuel's space were open at P6 in controls (**Figure 3i,m**) but failed to open in Cx26*^Sox10Cre^* mice (**Figure 3a–c,e–g**). Control cochleae from Cx26*^loxP/loxP^* mice presented with a time–dependent increase of connexin expression which was most evident in the spiral limbus, the sensory epithelium, and between the stria vascularis and the spiral ligament (**Figure 3i–p**) as previously reported [24]. In cochleae from Cx26*^Sox10Cre^* mice at P30, Cx26 and Cx30 were still present in the spiral limbus, spiral ligament (**Figure S1**) as well as in basal and intermediate cells of the stria vascularis (**Figure S2**). The latter finding is consistent with the residual endocochlear potential measured in Cx26*^Sox10Cre^* mice (**Figure 1c**).

### Characterization of gap junction channel permeability in the developing cochlea by fluorescence recovery after photobleaching

**Figure 4:**
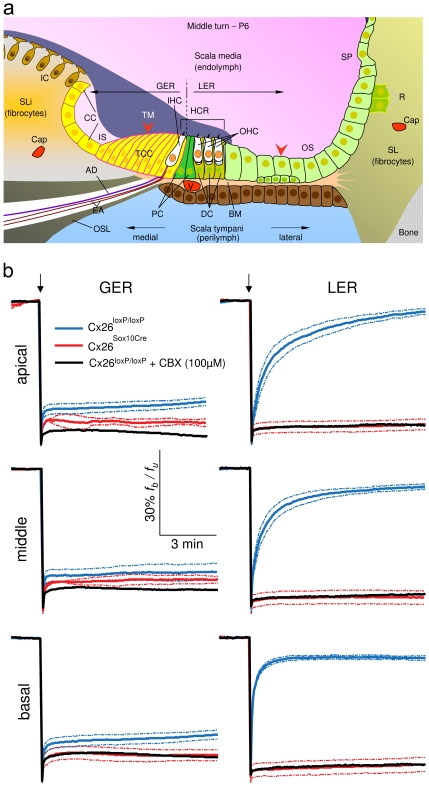
Dye coupling through gap junction channels in the developing cochlea of Cx26*^loxP/loxP^* and Cx26*^Sox10Cre^* mice. (**a**) Scheme of the sensory epithelium (midmodiolar section) in the developing cochlea (middle turn, P6); red arrowheads indicate the approximate position of laser foci in the receding greater epithelial ridge (GER) and in the lesser epithelial ridge (LER); AD, afferent dendrites of type I spiral ganglion neurons; BM, basilar membrane; Cap, capillary; CC, cuboidal cells; DC, Deiters' cells; EA, efferent axons; IC, interdental cells; IHC, inner hair cell; IS, inner sulcus; OHC, outer hair cells; OS, outer sulcus; OSL, osseous spiral lamina; PC, pillar cells; R, root cells; SL, spiral ligament; SLi, spiral limbus; SP, spiral prominence; TCC, tall columnar cells; V, vas spiralis. (**b**) Plots of f_b_/f_u_ (bleached over unbleached fluorescence intensity) versus time in P5 organotypic cultures from the basal, middle and apical turns of the cochlea (see Materials and Methods). Solid lines are averages of n = 3 independent experiments, dashed lines indicate standard error of the mean. Downward arrows mark the time of laser pulse delivery. Carbenoxolone (CBX) is a non–specific inhibitor of gap junction channels [32].

In order to determine whether the hearing loss in Cx26*^Sox10Cre^* mice may be ascribed to a diminished cell–cell coupling during the crucial post–natal period, as observed in Cx30^(−/−)^ mice [25] and Cx30T5M knock in mice [26], we performed fluorescence recovery after photobleaching assays [27] in cochlear cultures obtained from P5 mice. In particular, we focused on non–sensory cells of the receding greater epithelial ridge, the region of the sensory epithelium that gives rise to the inner hair cells and medial non–sensory cells [28], [29]. We also measured coupling among non–sensory cells in the lesser epithelial ridge, the area thought to give rise to the outer hair cells and lateral non–sensory cells [28], [29] (**Figure 4a**). After overnight incubation *in vitro*, cochlear organotypic cultures were loaded with the acetoxymethyl ester of calcein, a fluorescent tracer that diffuses through gap junction channels in this preparation [19]. Following the delivery of a 405 nm laser pulse to a restricted tissue area, the intracellular calcein fluorescence was partially restored via diffusion of the indicator dye through gap junction channels from adjacent unbleached cells in Cx26*^loxP/loxP^* control cultures (**Figure 4b** **, blue traces**). Incomplete recovery of fluorescence intensity is ascribed to the fraction of the calcein pool which is not available for intercellular transfer (immobile fraction) due to trapping into subcellular organelles and/or binding to subcellular structures [30]. These experiments confirm that non–sensory cells in the normal developing cochlea are dye–coupled in all cochlear turns [31]. The different time courses of fluorescence traces in the lesser epithelial ridge (**Figure 4b** **, right**) compared with the greater epithelial ridge (**Figure 4b** **, left**) is indicative of a substantially larger immobile fraction in the latter. Targeted ablation of Cx26 in Cx26*^Sox10Cre^* cultures, and the consequent downregulation of Cx30, caused a substantial reduction of dye coupling levels in the greater epithelial ridge and a complete loss of dye coupling in the lesser epithelial ridge (**Figure 4b** **, red traces**). The process of fluorescence recovery after photobleaching was inhibited by pre–incubating Cx26*^loxP/loxP^* cochlear cultures for 20 min with 100 µM carbenoxolone, a broad spectrum inhibitor of connexin channels [32] (**Figure 4b** **, black traces**). The minute residual downward peak, which is resistant to carbenoxolone, is due to a small and rapid recovery of fluorescence caused by the diffusion of dye within the cell from deeper, relatively unbleached regions to more superficial, bleached regions.

### Connexin gene delivery to cochlear organotypic cultures by BAAV

**Figure 5:**
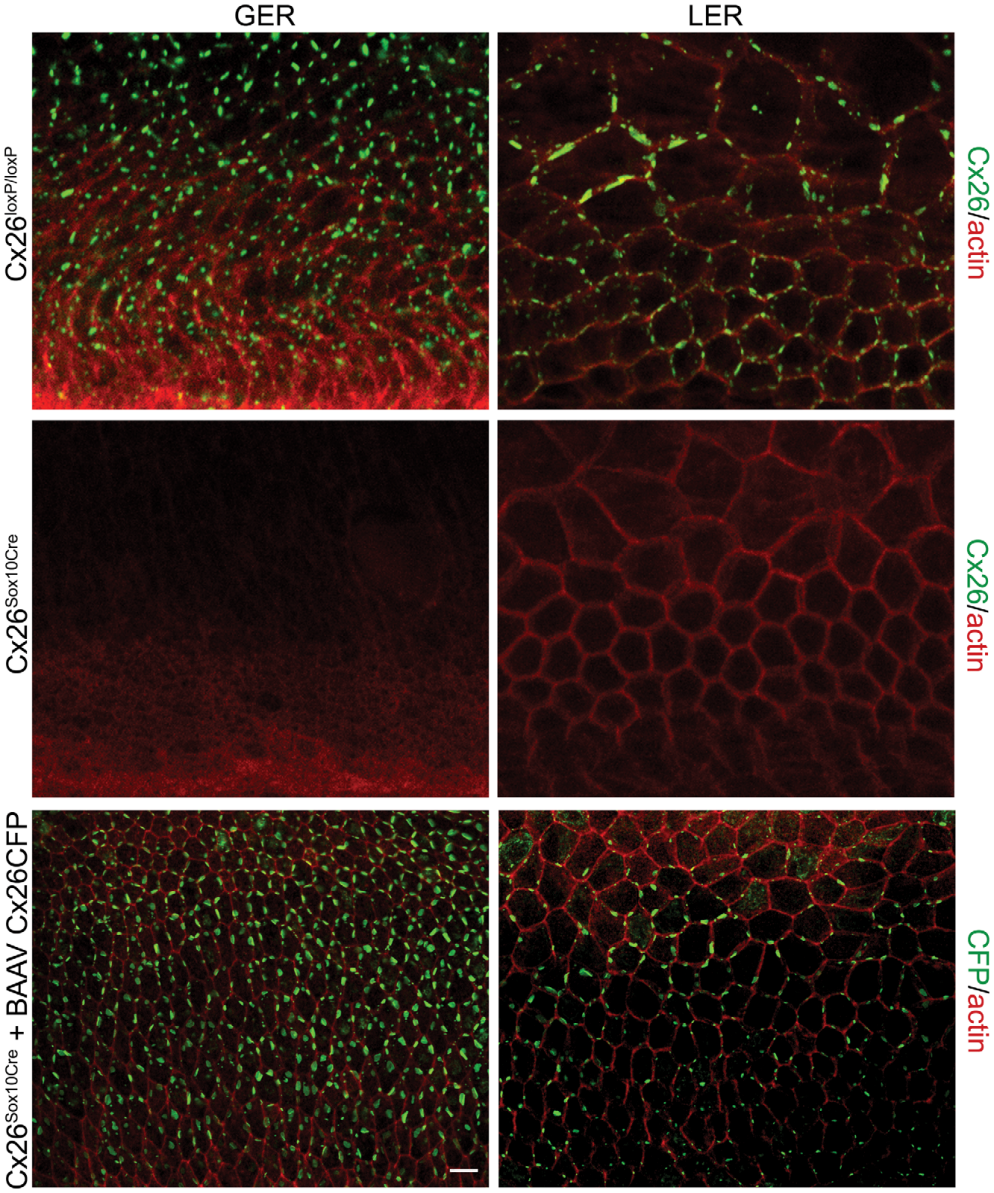
Recovery of Cx26 expression in Cx26*^Sox10Cre^* organotypic cultures transduced with BAAVCx26CFP. Top, immunoreactivity to Cx26 antibodies in a representative Cx26*^loxP/loxP^* culture. Middle, lack of Cx26 immunoreactivity in a representative Cx26*^Sox10Cre^* culture. Bottom, immunoreactivity to GFP antibodies, which also recognize CFP, in a representative Cx26*^Sox10Cre^* culture transduced with BAAVCx26CFP. In all panels, actin filaments were stained with Texas red conjugated phalloidin (red). Scale bar, 10 µm.

**Figure 6:**
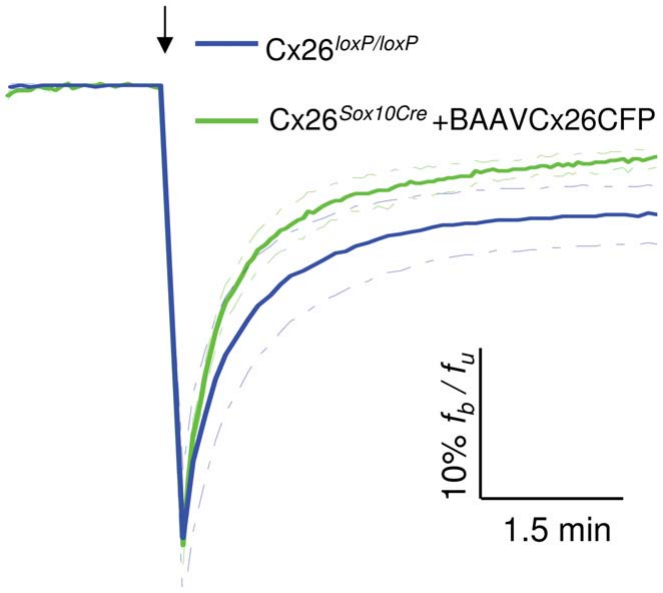
Rescue of dye coupling in Cx26*^Sox10Cre^* organotypic cultures transduced with BAAVCx26CFP. Plots of f_b_/f_u_ (bleached over unbleached fluorescence intensity) versus time in organotypic cultures from the middle cochlear turn. Solid lines are averages of n = 3 independent experiments, dashed lines indicate standard error of the mean. Downward arrow marks the time of laser pulse delivery.

We have previously shown that transduction with a BAAV vector encoding a Cx30GFP fusion protein (BAAVCx30GFP) restored gap junction coupling and intercellular Ca^2+^ signaling among non–sensory cells of Cx30^(−/−)^ organotypic cultures [25]. To test whether the expression level of Cx26 could be similarly restored in cochlear organotypic cultures obtained from Cx26*^Sox10Cre^* mice at P5, we used a BAAV vector encoding a Cx26CFP fusion protein (BAAVCx26CFP). Confocal fluorescence microscopy images obtained 48 hours post transduction showed recombinant Cx26CFP protein expressed in a large fraction of the non–sensory cells (**Figure 5**). The recombinant protein expression pattern (**Figure 5** **, bottom**) resembled closely that of endogenous Cx26 in control cultures from Cx26*^loxP/loxP^* mice immunoassayed with a Cx26 specific antibody (**Figure 5** **, top**) whereas no Cx26 immunoreactivity was detected in untreated Cx26*^Sox10Cre^* cultures (**Figure 5** **, middle**) consistent with the results shown in **Figure 3a–d**. Moreover, recovery of fluorescence after photobleaching of calcein in Cx26*^Sox10Cre^* cultures transduced with BAAVCx26CFP was even faster than that of untreated Cx26*^loxP/loxP^* control cultures (**Figure 6**) possibly due to a higher-than-normal level of recombinant Cx26 expression driven by the CMV promoter in the BAAV vector.

## Discussion

Gene therapy offers an attractive method for modulating gene expression in the inner ear with the ultimate goal of treating cochlear disorders. Recombinant BAAV vectors have several attributes that make them well suited for gene transfer in the inner ear, including efficient gene transfer *in vivo* and *in vitro*, low toxicity, and a unique serological identity. The tropism for the inner ear has been demonstrated by several groups and appears to be well tolerated [23], [25], [33], [34]. Indeed, in our study we report transduction of most of the non-sensory cells in cochlear organotypic cultures (**Figure 5**). *In utero*, apical transduction has also been reported which would be preferred in an *in vivo* gene therapy application [33]. Cell surface receptors are an important determinant of vector transduction and characterization of BAAV transduction requirements have demonstrated sialic acid containing gangliosides are essential for BAAV entry and transduction [35], [36], and multiple gangliosides are expressed in the mammalian cochlea [37], [38], [39].

Auditory threshold measurements indicate that Cx26*^Sox10Cre^* mice were severely deaf, despite the presence of a residual endocochlear potential of about 40 mV (**Figure 1**). Deafness in these mice was accompanied by degeneration of the organ of Corti in the basal cochlear turn and by a gradient of hair cell loss along the coiling axis of the cochlea (**Figure 2**). Prior in situ hybridization work in the postnatal mouse cochlea detected Sox10 mRNA expression in the supporting cells of the organ of Corti, namely inner sulcus cells, inner pillar cells, Deiters' cells, Hensen's cells, Claudius' cells and outer sulcus cells, but not in sensory hair cells and in cells of the stria vascularis [22]. Consistent with these findings, we report complete Cx26 ablation in the epithelial gap junction network of the cochlea, from the spiral limbus to the spiral prominence (**Figure 4a**) including the organ of Corti (**Figure 3a–d** and **Figure S1**). Furthermore, Cx30 immunofluorescence highlighted a delayed development of the labeling pattern in cells forming the epithelial gap junction network (**Figure 3e–h**). In the basal turn of the cochlea, the tunnel of Corti and Nuel's space were open at P6 in controls (**Figure 3i,m**) but failed to open before degeneration of the organ of Corti in Cx26*^Sox10Cre^* mice (**Figure 3a–h**) indicative of developmental defect.

Functional gap junction channels are crucial for maturation of different tissues [40]. Furthermore, several lines of experiments indicate that permeability to larger metabolites, rather than small inorganic ions, may play an important role in the development, physiology and aetiology of connexin–related diseases [41]. Our experiments of calcein fluorescence recovery after photobleaching showed reduced cell–cell communication in the epithelial network of Cx26*^Sox10Cre^* mice (**Figure 4b**). Altogether, these results indicate that ablation of Cx26 is associated with altered expression of Cx30 in the organ of Corti and affects not only hair cell survival but also the normal development of the organ of Corti.

Cx26 and Cx30 may assemble to form heteromeric and heterotypic intercellular channels [9], which mediate the transfer of ions, metabolites and second messengers, including inositol 1,4,5–trisphosphate (IP_3_) between cochlear non–sensory cells [42], [43]. Extrajunctional connexons, also referred to as connexin hemichannels, have been implicated in the release of signaling molecules, such as ATP, to the extracellular medium and proposed to serve a variety of paracrine signaling roles [44]. Connexins in the sensory epithelium of the inner ear form hemichannels that release ATP to the extracellular medium [45] and thus sustain intercellular Ca^2+^ signal propagation [19], [31]. Recently, studies performed in a Cx30T5M knock in mouse model linked hearing loss to a diminished frequency of spontaneous Ca^2+^ transients, mediated by ATP release through connexin hemichannels in the developing cochlea [26]. Prior work performed on cochlear organotypic cultures from Cx30^(−/−)^ mice highlighted a Ca^2+^–dependent coordinated regulation of Cx26 and Cx30 expression mediated by signaling through phospholipase C and the NF–κB pathway [25].

Recent work has suggested that lateral wall gap junction channels interconnecting cells of the spiral ligament and stria vascularis acquire mature features as early as P7 in the rat [46]. The time course of immunofluorescence labeling in the lateral wall of Cx26*^Sox10Cre^* mice showed no appreciable differences in the expression of either connexins with respect to control Cx26*^loxP/loxP^* mice (**Figure S2**). These data suggest that intercellular communication is preserved in the connective tissue network of Cx26*^Sox10Cre^* mice and that the reduced endocochlear potential (**Figure 1c**) is likely ascribed to morphological abnormalities in the sensory epithelium. Indeed, endocochlear potential values developed normally up to P12–P13 in the related Cx26*^OtogCre^* mouse model, decreasing significantly shortly after the onset of hearing in parallel with the appearance of epithelial breaches that compromised the integrity of the endolymphatic compartment [14].

Cx26*^OtogCre^* mice were obtained by crossing Cx26^l*oxP/loxP*^ mice with OtogCre founder mice, generated by pronuclear injection of the OtogCre BAC insert into C57BL/6 oocytes, expressing the Cre recombinase under the control of the murine Otogelin promoter. The deafness phenotype was more exacerbated in our Cx26*^Sox10Cre^* mice than in Cx26*^OtogCre^* mice, which presented with progressive and significant hearing loss ranging from 30 dB to 70 dB [14]. These differences may depend on the different techniques adopted to create their respective deleter mouse lines, and/or on specific features of the Otogelin and Sox10 promoters in the inner ear despite the overlapping expression patterns.

Hearing loss in our Cx26*^Sox10Cre^* mice was comparable to that of a conditional Cx26 null mouse model (cCx26) obtained by crossing Cx26*^loxP/loxP^* mice with Rosa26–Cre^ERT^ mice, in which Cre can be activated by a single injection, on embryonic day 19, of the synthetic estrogen 4–hydroxytamoxifen [18]. However, peripheral nerve fibers and the somata of spiral ganglion neurons at corresponding cochlear locations were completely degenerated in cCx26 mice, whereas there was no sign of degeneration in spiral ganglion neurons of Cx26*^OtogCre^* mice [14] or Cx26*^Sox10Cre^* mice (**Figure S3**).

Transduction of Cx26*^Sox10Cre^* cochlear organotypic cultures with BAAV at P5 not only restored Cx26CFP correctly targeted to the plasma membrane of cochlear non–sensory cells (**Figure 5**) but also induced the formation of functional intercellular channels at points of contact between adjacent cells (**Figure 6**). BAAV [47] exhibits preferential tropism for cochlear non–sensory cells, with sporadic transduction of sensory inner and outer hair cells [23], [25], [33], [34]. These results suggest that restoration of normal connexin levels by gene delivery via recombinant AAV could be a way to rescue hearing function in DFNB1 mouse models and might, in future, lead to the development of therapeutic interventions in humans, particularly in children.

## Materials and Methods

### Transgenic mice and genotyping

Animal handling was approved by the Ethical Committee of Padua University (Comitato Etico di Ateneo per la Sperimentazione Animale, C.E.A.S.A.) project n. 54/2009, protocol n. 51731. The background strains of the transgenic mice used in this study were: (a) Cx26*^loxP/loxP^* [14], mixed C57BL/6 and 129/SvPasCrl; (b) Sox10CRE [20], mixed BL6CBAF1 and 129/Sv. Double transgenic Cx26*^Sox10Cre^* mice were detected by screening for the presence of the two insertions, *loxP* and *Cre*, by PCR on extracted mouse tail tips using the following primers (see also Suppl. Mat. in Ref. [14]):

Cx26F 5′–TTTCCAATGCTGGTGGAGTG–3′
Cx26R 5′–ACAGAAATGTGTTGGTGATGG–3′
CreF 5′–CATTACCGGTCGATGCA–3′
CreR 5′–GAACCTGGTCGAAATCAG–3′.

Cre recombinase transmitted via maternal germline was activated only in Sox10 expressing cells whereas transmission via paternal germline ensued in early Cre activation in the whole embryo and consequent lethality.

### In vivo recordings of endocochlear potential and auditory brainstem responses

To measure endocochlear potential, mice were anaesthetized with 0.01 ml/g body weight of 20% urethane, a tracheal cannula was inserted, and the bulla opened to reveal the cochlea whilst body temperature was kept at 38°C by a feedback–controlled heating pad. A small hole was made in the bony wall of the cochlea over the basal turn scala media, and a micropipette electrode filled with 150 mM–KCl was advanced through the hole and through the spiral ligament of the lateral wall into the scala media. The potential difference between the scala media and a reference silver/silver chloride pellet under the dorsal skin was recorded [48].

To record auditory brainstem responses, mice were anesthetized with an intraperitoneal injection of zolazepam (25 µg/g) and xylazine (10 µg/g). Supplemental doses were then administered as needed whilst body temperature was kept at 38°C by a feedback–controlled heating pad. Each recording procedure lasts up to 70 min. Acoustic stimuli were produced in the free field within a foam–padded, shielded acoustic chamber by a System 3 Real–time Signal Processing System combined with an ES1 electrostatic speaker (Tucker–Davis Technologies, Alachua, FL, U.S.A.) positioned 4 cm lateral to the left ear of the mouse [49]. Stimuli were calibrated by means of a ECM8000 measurement microphone (Behringer International GmbH, Willich, Germany) mounted on the 800B Larson–Davis sound level meter and placed in the position to be occupied by the mouse ear. Stimuli consist of tone bursts (1 ms rise/decay; 3 ms plateau) at 8, 14, 20, 26, and 32 kHz, and clicks (0.1 ms) delivered at a repetition rate of 13 Hz. A maximum peak equivalent sound pressure level (SPL) of 100 dB (re: 20 µPa) was employed for clicks as well as tone bursts. Decreasing SPLs of 10 dB were employed, starting from a maximum of 100 dB SPL. To minimize contralateral acoustic stimulation, the outer ear canal of the right ear was filled with condensation–vulcanizing silicone mixed with hardener paste (Otoform A flex, in double cartridges, Dreve Otoplastik, Unna, Germany) delivered through a mixing cannula (diameter 5.4 mm) and dispensed by an injector (DS50, Dreve Otoplastik). Acquisition and analysis time was 12 ms for each single stimulus. Responses were recorded between subcutaneous needle electrodes inserted at the vertex (active) ventrolateral to the left ear (reference) and above the tail (ground). Potential differences were amplified (×50000) with an isolated instrumentation amplifier, band pass filtered below 100 Hz and above 8000 Hz and digitized at a rate of 40000 samples per second. Response waveforms were typically obtained from averages of 400 stimuli presented at the rate of 13 per second for each stimulus condition, saved in non volatile memory of a computer and finally displayed on a computer screen.

LabVIEW software (version 8.0.1, National Instruments, Austin, TX, U.S.A.) was used for measurements and analysis of amplitude and latency of auditory brainstem responses. To reduce noise and abrupt transitions in the temporal domain, traces were smoothed digitally by low–pass finite impulse response (FIR) filtering with equi–ripple characteristics using the Parks–McClellan algorithm. Low–pass frequency was fixed two octaves below the original sampling frequency. For quality control, the smoothed trace was checked against the original trace on the computer display. Wave amplitudes were computed by a peak detection algorithm as the difference between the two values represented by response maxima (peak) and minima (valley). The algorithm fits a quadratic polynomial curve to sequential groups of data points (the number of data points used in the fit was 3). Peak latencies were determined relative to the onset of the acoustic stimulus. Wave IV was the most stable and robust evoked response at all intensity levels and for all types of stimulus. The corresponding peak was therefore utilized to estimate auditory brainstem response threshold, defined as the lowest sound pressure level at which any peak could be detected above the residual noise by an experimentally experienced observer blind with respect to genotype. If no wave was detected at maximum intensity stimulation, a nominal threshold of 110 dB SPL was assigned. If any wave was detected at minimum intensity stimulation of 1 or 10 dB SPL, a nominal threshold of the same level was assigned. Stimuli of lower intensities were not applied due to intrinsic limitations of the sound delivery system.

### BAAV production

Semiconfluent 293T cells were transfected by calcium phosphate with four required plasmids: transgene vector, pAd12, AAV2–Rep and BAAV–Cap. AAV–2 ITRs were used in the vector plasmid. Forty–eight hours post–transduction, cells were harvested by scraping in a solution containing 140 mM NaCl, 5 mM KCl, 0.7 mM K_2_HPO_4_, 25 mM Tris–HCl, pH 7.4 (TD buffer) and the cell pellet was concentrated by low–speed centrifugation. Cells were lysed in TD buffer containing 0.5% deoxycholate and 100 U/ml DNase (Benzonase, Sigma) and incubated for 30 min at 37°C. Following 10 min low speed centrifugation, the vector was purified using CsCl gradients. Particle titers were determined by quantitative polymerase chain reaction (qPCR) and biological activity was tested on Hek293T cells. For viral titration, a dilution of the viral preparation was added to a PCR mixture containing 1× SYBR Green Master Mix (Applied Biosystems/Applera) and 0.25 pmol/µl forward and reverse primers. Amplification was measured using a sequence detector (ABI 7700, Applied Biosystems). Specific primers for cytomegalovirus (CMV) were designed with the Primer Express program (Applied Biosystems):

CMV forward 5′–CATCTACGTATTAGTCATCGCTATTACCAT–3′,
CMV reverse 5′–TGGAAATCCCCGTGAGTCA–3′.

Following denaturation at 96°C for 10 min, cycling conditions were 96°C for 15 s, 60°C for 1 min for 40 cycles. The viral DNA in each sample was quantified by comparing the fluorescence profiles with a set of DNA standards. Titers were in the range of 10^12^–10^13^ BAAV particles/ml.

### Cochlear cultures and transduction with BAAV

Cochleae were dissected from P5 mouse pups in ice–cold Hepes buffered (10 mM, pH 7.2) Hanks' balanced salt solutions (HBSS, Invitrogen) and placed onto glass coverslips coated with 136 µg/ml of Cell Tak (Becton Dickinson). Cultures were incubated in Dulbecco's modified Eagle's medium supplemented with the F12 growth factor (DMEM/F12, Invitrogen) supplemented with 5% fetal bovine serum (FBS) and maintained at 37°C for 1 day. Transduction with viral constructs was performed by adding purified and dialyzed vector at a final titer of 10^11^ particles/ml in culture medium devoid of FBS. Cultures were kept in this medium at 37°C for the first 24 h, to favor viral transduction, and thereafter maintained in DMEM/F12 supplemented with FBS up to 48 h before experiments.

### Calcein measurements and fluorescence recovery after photobleaching

Focal irradiation of live cochlear cultures was used to photobleach calcein, as previously described [19], [25], [31]. Calcein is a polyanionic fluorescein derivative that exhibits fluorescence essentially independent of pH between 6.5 and 12. It has about six negative and two positive charges at pH 7 (net charge −4, MW 622) and permeates through gap junction channels in the immature organ of Corti [19]. In these experiments, the output of a semiconductor lased module (50 mW, 405 nm, part number LGT 405–60, LG–Laser Technologies GmbH) was injected into a UV permissive fiber optic cable (single mode 0.1 N.A., Mode Field Diameter (MDF) 3.2±0.5 µm, part number P1–405A–FC–2, Thorlabs GmbH); fiber output was collected through a collimating aspheric lens (5 mm effective focal length, part number HPUCO–23A–S–6.2AS, LG–Laser Technologies GmbH) and the re–collimated beam was directed onto a dichromatic mirror (440 dclp, Chroma Technology Corp.) placed at 45° just above the objective lens of the microscope. By carefully adjusting the position of the fiber in front of the aspheric lens with a two–axis micromanipulator (part number HPT1, Thorlabs) we projected a sharp image of the illuminated fiber core (spot) onto the specimen focal plane selected by the (infinity corrected) objective lens. Under these conditions, the fiber optic diameter determined accurately the laser irradiated area, which encompassed one to few cells, depending on cell size and location within the sensory epithelium [31].

For staining with calcein, live cultures were incubated for 10 min at 37°C in an extracellular medium (EXM) containing 138 mM NaCl, 5 mM KCl, 2 mM CaCl_2_, 0.3 mM NaH_2_PO_4_, 0.4 mM KH_2_PO_4_, 10 mM HEPES–NaOH and 6 mM d–glucose, pH 7.2 and 320 mOsm, supplemented with 5 µM calcein–AM, plus 250 µM sulphinpyrazone and 0.01 w/v pluronic F–127 to prevent dye sequestration and secretion [50]. For recording, cultures were transferred on the stage of an upright fluorescence microscope (BX51, Olympus Corporation, Tokyo, Japan) and perfused in EXM for 20 min at 2 ml/min to allow for de–esterification, and thereafter maintained in still EXM at room temperature. Calcein fluorescence was excited and detected using a U–MGFHQ filter cube (Olympus) incorporating a BP460–480 excitation filter, a DM485HQ dichromatic mirror and a BA495–540HQ barrier (emission) filter. Cultures were imaged with a 60× water immersion objective (0.90 NA, Lumplan FL, Olympus) and fluorescence emission was monitored with a cooled charge–coupled device (CCD) camera (Sensicam QE, PCO AG). In all experiments, the effects of photobleaching due to sample illumination in the 460–480 nm spectral window were kept under control by carefully selecting the most appropriate inter–frame interval (4 s) while controlling light exposure (50 ms) with a mechanical shutter triggered by the frame–valid (FVAL) signal of the CCD camera. Baseline fluorescence in the 495–540 nm emission window was recorded for 2 min, followed by focal laser irradiation at 405 nm to bleach intracellular calcein. Laser irradiation intervals were adjusted to cause 50% photobleaching of the mean baseline fluorescence, which required 0.5 s for cells of the greater epithelial ridge and 1.2 s for cells of the lesser epithelial ridge. Fluorescence recovery after photobleaching was monitored for up to 10 min. Image sequences were acquired using software developed in the laboratory, stored on disk and processed off–line using the Matlab 7.0 software package (The MathWorks, Inc.).

For the analysis of fluorescence recovery after photobleaching, we delineated a region of interest (ROI) inside the bleached (*b*) area, plus a ROI in a proximal unbleached (*u*) area, and we computed ratios of fluorescence intensities (*f_b_*/*f_u_*) at each time point, as described in Refs. [19], [25], [31].

### Immunohistochemistry and confocal imaging

Cochleae were dissected, fixed in 4% paraformaldehyde for 20 min at room temperature, rinsed in phosphate buffered saline (PBS) and decalcified over night in ethylenediaminetetraacetic acid (EDTA, 0.3 M). After 3 washes in PBS, preparations were included in 3% agarose dissolved in PBS and cut in 100 µm thickness steps using a vibratome (VT 1000S, Leica). Tissue slices were permeabilized for 3 hours at room temperature with 0.1% Triton X–100, dissolved in bovine serum albumin (BSA) 2% solution. A slightly different procedure was used for cochlear organotypic cultures, which were fixed for 15 min and permeabilized for 30 min. Connexins were immunolabeled by overnight incubation at 4°C with specific polyclonal rabbit anti–Cx30 antibodies (Invitrogen, Cat. No. 71–2200) and anti–Cx26 antibodies (Invitrogen, Cat. No. 51–2800) diluted in BSA 1% rinse solution (2.5 µg/ml). Organotypic cultures transduced with BAAVCx26CFP were instead immunolabeled with an anti–GFP antibody (Invitrogen, Cat. No. A11122) which also recognized CFP, in order to amplify the Cx26CFP signal. Tissues were then washed three times in PBS (each time for 1 h). Secondary antibodies (Alexa Fluor® 488 goat anti–rabbit IgG, Invitrogen, Cat. No. A–11008) were applied overnight at 5 µg/ml and room temperature to tissue slices (for 1 hour to organotypic cultures) whilst F–Actin was stained by incubation with Texas Red–X phalloidin (Invitrogen, Cat. No. T7471) and nuclei were stained with 4′,6–diamidino–2–phenylindole, dihydrochloride (DAPI, Invitrogen, Cat. No. D1306) both diluted in BSA 1% rinse solution (1∶200). After washing for further three times in PBS, samples were mounted onto glass slides with a mounting medium (FluorSave™ Reagent, Merk, Cat. No. 345789) and analyzed using a confocal microscope (TCS SP5, Leica) equipped with an oil–immersion objective (either 40× HCX PL APO 1.25 N.A., or 63× HCX PL APO 1.4 N.A., Leica). Laser line intensities and detector gains were carefully adjusted to minimize signal bleed through outside the designated spectral windows.

## Supporting Information

Figure S1 **Time course of connexin immunoreactivity in the cochlear duct.** Maximal projection rendering of two consecutive midmodiolar confocal optical sections taken at 1 µm intervals in the basal cochlear turn of Cx26*^Sox10Cre^* mice (a–h) and Cx26*^loxP/loxP^* mice (i–p) at P6, P9, P14 and P30. Expression of Cx26 (a–d, i–l) and Cx30 (e–h, m–p) was detected with selective antibodies (green) nuclei were stained with DAPI (blue) and actin filaments with Texas red conjugated phalloidin (red). Scale bars, 50 µm.
(TIF)

Figure S2 **Time course of connexin immunoreactivity in the cochlear lateral wall.** Maximal projection rendering of two consecutive midmodiolar confocal optical sections taken at 1 µm intervals in the basal cochlear turn of Cx26*^Sox10Cre^* mice (a–h) and Cx26*^loxP/loxP^* mice (i–p) at P6, P9, P14 and P30. Expression of Cx26 (a–d, i–l) and Cx30 (e–h, m–p) was detected with selective antibodies (green) nuclei were stained with DAPI (blue) and actin filaments with Texas red conjugated phalloidin (red). Scale bars, 50 µm.
(TIF)

Figure S3 **Confocal microscopy of spiral ganglion neurons in Cx26** ***^Sox10Cre^*** **mice at P30.** Nuclei were stained with DAPI (blue) and actin filaments with Texas red conjugated phalloidin (red). Scale bar, 25 µm.
(TIF)
