# Supplementary figures and images for: BAAV Mediated GJB2 Gene Transfer Restores Gap Junction Coupling in Cochlear Organotypic Cultures from Deaf Cx26Sox10Cre Mice
Source: PLoS One. 2011 Aug 18;6(8):e23279. doi: 10.1371/journal.pone.0023279 (PMC3158073; doi:10.1371/journal.pone.0023279)

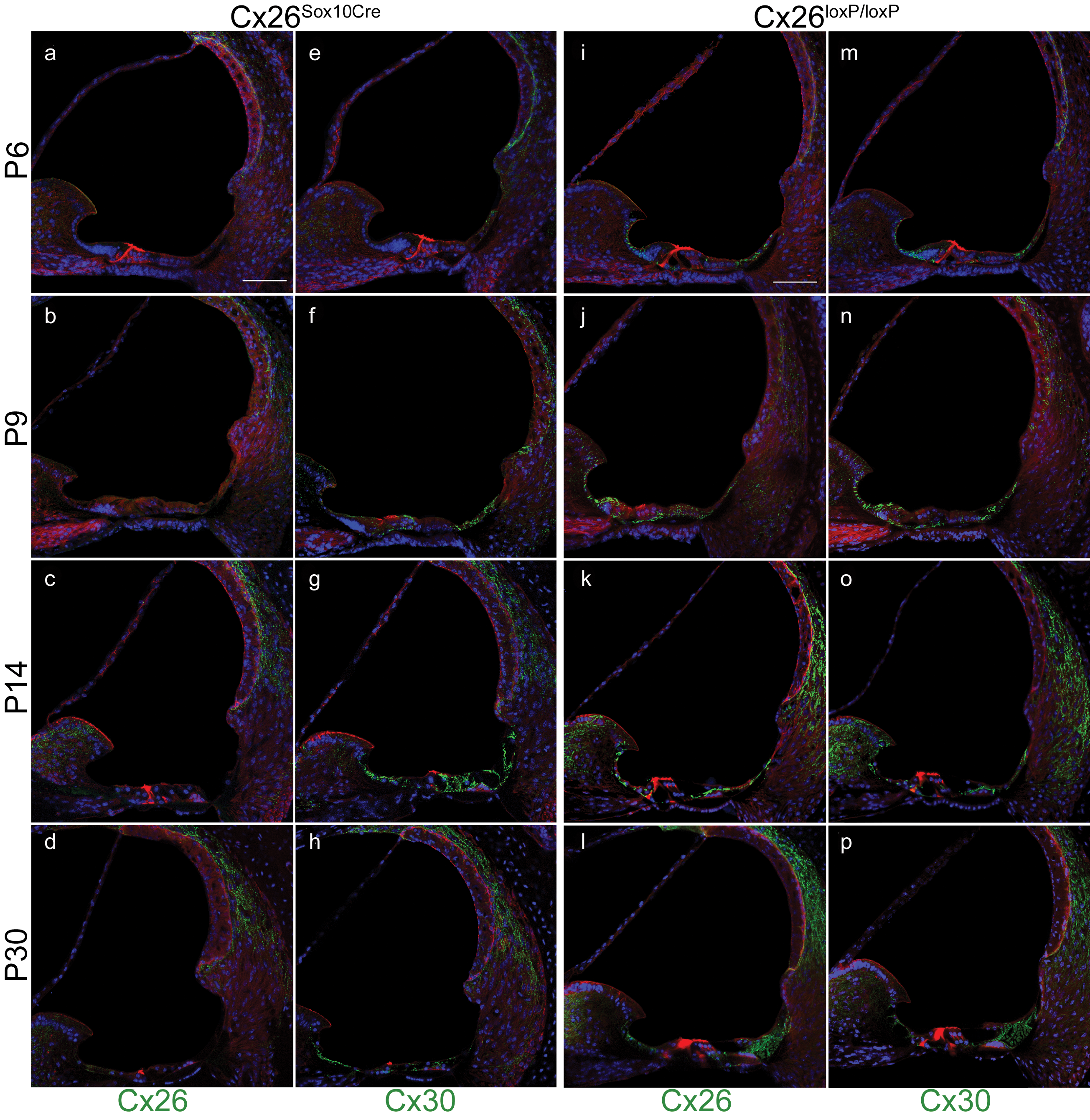

Supplement: Figure S1 — Time course of connexin immunoreactivity in the cochlear duct. Maximal projection rendering of two consecutive midmodiolar confocal optical sections taken at 1 µm intervals in the basal cochlear turn of Cx26Sox10Cre mice (a–h) and Cx26loxP/loxP mice (i–p) at P6, P9, P14 and P30. Expression of Cx26 (a–d, i–l) and Cx30 (e–h, m–p) was detected with selective antibodies (green) nuclei were stained with DAPI (blue) and actin filaments with Texas red conjugated phalloidin (red). Scale bars, 50 µm. (TIF) [file pone.0023279.s001.tif]

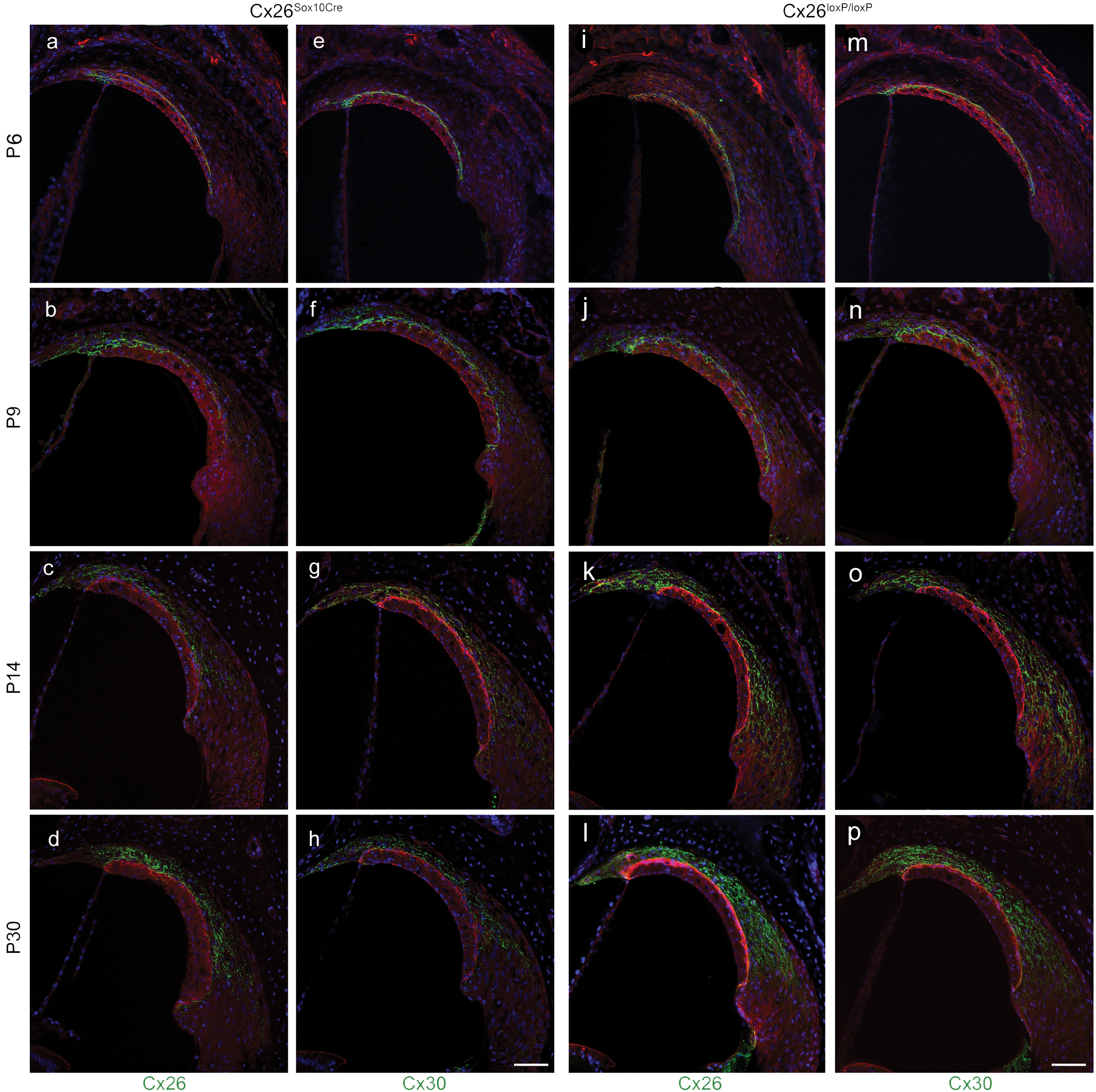

Supplement: Figure S2 — Time course of connexin immunoreactivity in the cochlear lateral wall. Maximal projection rendering of two consecutive midmodiolar confocal optical sections taken at 1 µm intervals in the basal cochlear turn of Cx26Sox10Cre mice (a–h) and Cx26loxP/loxP mice (i–p) at P6, P9, P14 and P30. Expression of Cx26 (a–d, i–l) and Cx30 (e–h, m–p) was detected with selective antibodies (green) nuclei were stained with DAPI (blue) and actin filaments with Texas red conjugated phalloidin (red). Scale bars, 50 µm. (TIF) [file pone.0023279.s002.tif]

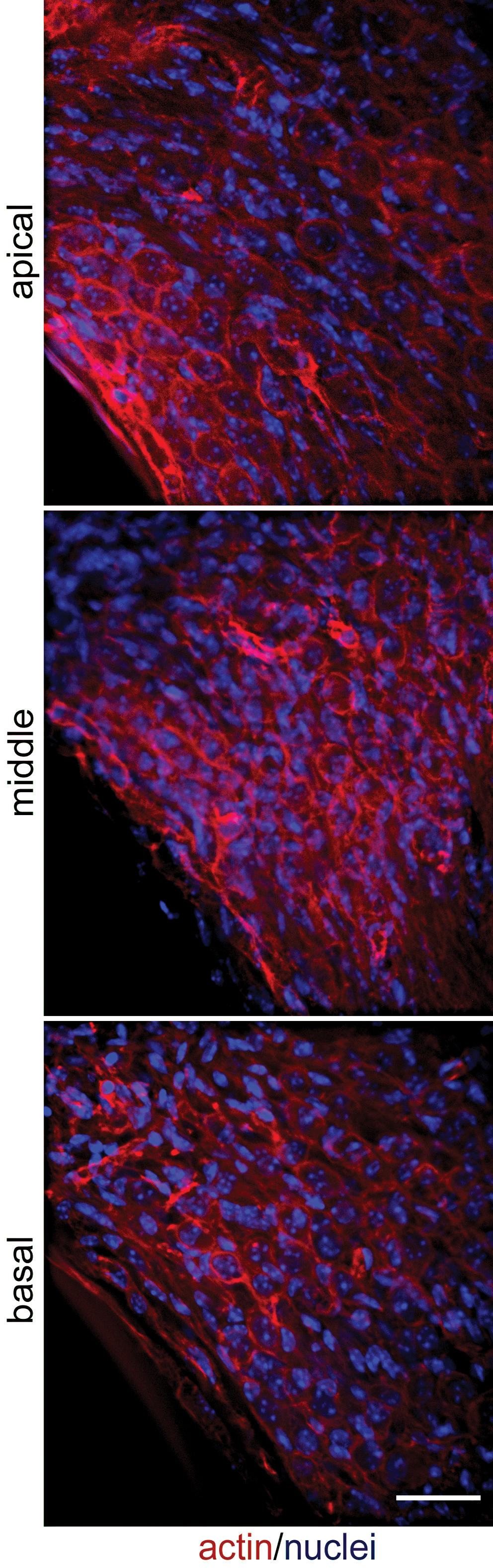

Supplement: Figure S3 — Confocal microscopy of spiral ganglion neurons in Cx26 Sox10Cre mice at P30. Nuclei were stained with DAPI (blue) and actin filaments with Texas red conjugated phalloidin (red). Scale bar, 25 µm. (TIF) [file pone.0023279.s003.tif]
